# Gender differences in the association between physical frailty and cognitive function among older adults: A cross-sectional study in rural Guizhou, China

**DOI:** 10.1038/s41598-026-54308-3

**Published:** 2026-05-22

**Authors:** Yongchun Hong, Ji Tang, Haiwen Qiu, Jingyuan Yang, Weifang Liao

**Affiliations:** 1 Clinical Medical College, Affiliated Hospital of Jiujiang University, Jiujiang, Jiangxi China; 2 Jiujiang Clinical Precision Medicine Research Center, Jiujiang, Jiangxi China; 3 Department of Neonatology, Shanghai Children’s Medical Center GuiZhou Hospital, Shanghai Jiao Tong University School of Medicine, Guiyang, China; 4 Department of Epidemiology and Health Statistics, School of Public Health, The Key Laboratory of Environmental Pollution Monitoring and Disease Control, Guizhou Medical University, Guiyang, China

**Keywords:** Physical frailty, Cognitive function, Gender, Older people, Diseases, Health care, Medical research, Risk factors

## Abstract

To examine gender differences in the association between physical frailty and cognitive function among older adults in rural China. A total of 1,654 older adults (41.9% male) aged 60 years and above from rural areas of Guizhou Province, China, participated in the study. Cognitive function was assessed using the Chinese-adapted Mini-Mental State Examination (C-MMSE), and frailty status was determined based on Fried’s frailty criteria. Multivariate linear regression analyses were conducted to examine the association between frailty and cognitive function, with sex-stratified analyses performed to explore gender-specific differences in these associations. Females exhibited a significantly higher prevalence of frailty (12.9% vs. 9.2%) and lower mean C-MMSE scores (19 vs. 24) compared to males (All *P* < 0.001). After adjustment, frailty was inversely associated with C-MMSE in both sexes.Among the five frailty phenotypes, only low physical activity (β =-1.156; 95% CI: -2.019 to -0.293; *P* = 0.002) and self-reported exhaustion or fatigue (β = -0.963; 95% CI:-1.658 to -0.269; *P* = 0.005) were significantly associated with lower C-MMSE scores in females. In women, the presence of any one or more frailty phenotypes was linked to cognitive decline, whereas in men, cognitive impairment was observed only when three or more frailty phenotypes were present. According to results from the study of older adults in rural Guizhou, there is a gender difference in the relationship between frailty and cognitive function, with women having a stronger association. These findings suggest that women’s cognitive function is more susceptible to the effects of frailty, while more prospective research is needed to confirm this conclusion.

## Introduction

The progressive aging of the global population is associated with the growing prevalence of dementia, imposing a substantial burden on families and society as a whole^1^. Effective treatments for dementia have yet to be established, emphasizing the need to identify risk factors associated with cognitive decline to facilitate early intervention-based preventative efforts^2^. Frailty has been repeatedly demonstrated to be closely related to the risk of cognitive decline and dementia, with frail older adults being more likely to experience cognitive decline^3–6^. Frailty is a dynamic and reversible physical state, and interventional strategies targeting frailty may also help decrease the risk of cognitive decline and dementia^7–11^.

Frailty is one of the most common syndromes observed in older adults and is characterized by aging-related reductions in physical reserves and vitality^12,13^. Frailty has been defined as consisting of five different biological components, including weakness, exhaustion, slow gait speed, unintentional weight loss, and low physical activity levels^14^. Some of the frailty-related phenotypes, such as slow gait speed, lower grip strength and impaired motor skills, have been reported to be associated with risk of cognitive decline. Prevalence of frailty and cognitive impairment in females have also been noted to be significantly higher than those in males^15–17^. The phenotypic components of frailty also vary by gender, with older men more likely than women to experience unintentional weight loss and slow gait^16,18^. Studies found differences in specific areas of cognitive function and Alzheimer’s disease risk, with women showing greater verbal and object localization skills, while men showed greater spatial memory skills^19^. Gender differences in the prevalence of cognitive impairment in older adults were also observed^20^. prior studies have shown that gender has a significant impact on the prevalence of frailty and cognitive functioning in older adults, and it is hypothesized that gender may also act as an important moderator or influencer, influencing the association between frailty and cognitive functioning in older adults. However, few studies have focused specifically on gender differences in the relationship between cognitive ability and overall frailty or individual components of frailty.

Exploring gender differences between frailty and cognitive function may provide a more accurate basis for gender-specific interventions and an evidence-based basis for risk stratification of older adults to prevent dementia. Here, we conducted a cross-sectional analysis of a population of older adults in a rural community in Guizhou Province, China, aiming to directly analyze potential gender differences between frailty and cognitive performance.

## Methods

### Study design and participants

The present cross-sectional epidemiological analyses utilized baseline data pertaining to the health status of older adults from the rural Guizhou region of China (SHGROC), with sampling details having been reported previously^21^. Briefly, this study was conducted in the rural Guizhou Province in southwestern China from July 15 – August 3, 2019, using a multi-stage cluster random sampling approach. Initially, two rural towns in this province were selected in two random counties, after which several villages from these towns were randomly selected, ultimately leading to the inclusion of 1,654 adults over 60 years of age in the final survey. To be eligible for inclusion, participants had to (1) be ≥ 60 years old and have resided in this rural area for at least 5 years, (2) exhibit normal verbal communication abilities, and (3) be capable of self-managing their daily life without any self-recognized cognitive dysfunction. Participants were excluded if they (1) had severe mental or physical disorders or had been diagnosed with dementia, or (2) if they were not able to participate in the survey.

### Ethical considerations

The medical ethics committee of Guizhou Medical University approved this study(approval No.2018-092), and all participants provided written consent after having been informed of the purpose of the survey. All methods in this study were performed in accordance with the guidelines of the Declaration of Helsinki.

### Assessment of cognitive function

Cognitive function was assessed using the Chinese-adapted version of the Mini-Mental State Examination (C-MMSE), which has been culturally and linguistically validated for use in Mainland China^22^.Trained investigators administered the C-MMSE in Chinese and recorded participants’answers following standardized instructions. Total scores range from 0 to 30, with higher scores indicating better cognition. In this study, we analyzed C-MMSE total scores as continuous outcomes. No original C-MMSE/MMSE items, stems, or verbatim content are reproduced in the manuscript; only summary scores are reported, with appropriate citation to the Chinese adaptation.

### Assessment of frailty

Previously reported criteria were used to assess the frailty status of these patients^23^. Participants were considered frail if they exhibited three or more of the following: (1) unintended weight loss of at least 4.5 kg or 5% of body weight over the past 12 months, (2) low handgrip strength after adjusting for gender and BMI, (3) self-reported exhaustion or fatigue, (4) slower movement, as measured based on the time required to walk 4.6 m with adjustments for height and gender, and (5) low physical activity levels relative to a threshold of 3 h of activity per week and adjusted for gender.

### Covariates measurement

Trained interviewers administered a self-developed questionnaire to all participants and conducted corresponding physical examinations. Items included on the developed questionnaire included the following: (1) Demographic characteristics (age, gender, education, marital status, and annual household income), (2) Lifestyle characteristics (smoking status, drinking status, and physical exercise), (3) Hobbies (including reading, watching TV, gardening, playing chess, and playing cards), (4) History of chronic disease based on either a self-reported diagnosis, a diagnosis in higher-level hospitals, or lifelong symptoms, (5) Sleep quality over the past months as measured with the 8-item Athens Insomnia Scale (AIS)^24^, (6) Depression screening results determined using two questions from the Patient Health Questionnaire-2 (PHQ-2), with a score ≥ 3 being consistent with depression^25^, (7) The revised Social Support Rating Scale (SSRS) produced by Xiao et al., which included 10 items including 3, 4, and 3 respectively related to objective support, subjective support, and support utilization such that low, intermediate, and high grades were applied to scores of ≤ 22, 23–44, and > 45, respectively^26^, and (8) BMI, which was stratgified into three groups based on Chinese standards (< 18.5, 18.5–23.9, and ≥ 24 kg/m^2^)^27^.

### Statistical analysis

Non-normal continuous data (C-MMSE scores) were statistically described using median and interquartile ranges, and categorical variables were statistically described using proportions or rates. The *χ*²test was used to compare statistical differences between categorical variables, and the Mann-Whitney U test was used for continuous measures. Multiple linear regression was used to assess the association between physical decline and cognitive function, β coefficient values were reported with 95% confidence intervals (CIs). Model 1 was analyzed using univariate analyses, while Model 2 was adjusted for age, education, marital status, chronic disease, physical activity, depression, sleep quality, and body mass index. Subgroup analyses were performed after stratification by sex to assess sex differences in the relationship between physical frailty and the number of positive components and cognitive function. All statistical tests were performed using SPSS 25.0, and a two-sided *P* < 0.05 was considered statistically significant.

## Results

A total of 1654 subjects were included in this study for analysis, with a mean age of 71.4 ± 6.5 years (range: 60–96 years), including 693 men (41.9%, 70.7 ± 6.3 years) and 961 women (58.1%, 71.9 ± 6.7 years). There were significant differences between male and female participants in terms of household income and frailty. Women also had a significantly higher prevalence of frailty (12.9% vs. 9.2%) and tended to have significantly lower C-MMSE scores (19 vs. 24) (both *P* < 0.001) (Table 1).

**Table 1:** Baseline characteristics of male and female participants.

Characteristics	Total (*n*%) *n* = 1654	Men [*n* = 693(41.9%)]	Women [*n* = 961(58.1%)]	*P*-Value^a^
Age, mean ± SD	71.38 ± 6.55	70.68 ± 6.34	71.88 ± 6.65	< 0.001
Illiterate	1271(76.8)	408(58.9)	863(89.8)	< 0.001
Married	1013(61.2)	527(76.0)	486(50.6)	< 0.001
Annual household income				0.711
<10,000 Yuan	549(33.2)	224(32.3)	325(33.8)	
10,000-Yuan	520(31.4)	225(32.5)	295(30.7)	
≥ 30,000 Yuan	585(35.4)	244(35.2)	341(35.5)	
Chronic disease	1039(62.9)	408(58.9)	631(65.7)	0.005
Current smoke	450(27.2)	424(61.2)	26(2.7)	< 0.001
Current drinker	438(26.5)	309(43.6)	136(14.2)	< 0.001
Physical exercise (>30 min/d)	726(43.9)	307(44.2)	420(43.7)	0.855
Depression	632(38.2)	214(30.9)	418(43.5)	< 0.001
Sleep quality, mean ± SD	4.55 ± 4.39	3.92 ± 4.05	5.00 ± 4.57	< 0.001
Good	885(53.6)	415(59.9)	470(48.9)	
Medium	283(17.1)	113(16.3)	170(17.7)	
Poor	484(29.3)	164(23.7)	320(33.3)	
Social support lever, mean ± SD	34.75 ± 6.86	35.82 ± 6.87	33.97 ± 6.75	< 0.001
Low level	79(4.9)	23(3.3)	56(5.8)	
Medium level	1444(87.3)	595(85.9)	849(88.3)	
High level	128(7.7)	72(10.8)	53(5.5)	
BMI(kg/m^2^), mean ± SD	22.46 ± 3.53	22.24 ± 3.36	22.60 ± 3.65	0.007
<18.5	172(10.4)	65(9.4)	107(11.1)	
18.5–23.9	933(56.4)	417(60.2)	516(53.7)	
≥ 24	485(29.3)	177(25.5)	308(32.0)	
Frailty	213(12.9)	64(9.2)	149(15.5)	< 0.001
MMSE score, (M, Q_L_∼Q_U_)	21(17 ~ 25)	24(20 ~ 27)	19(16 ~ 23)	< 0.001

*Abbreviations*: BMI: body mass index; SD: standard deviation; MMSE: Mini-Mental State Examination.

^a^ Independent t-test for continuous variables and chi-square test for categorical variables.

C-MMSE scores by gender and demographic characteristics are shown in Table S1. There was a decreasing trend in C-MMSE scores with increasing age and an increasing trend in C-MMSE scores with increasing levels of education, annual household income, and social support (all *P* < 0.001). Participants with lower cognitive scores tended to be older, female, illiterate, unmarried, and depressed (all *P* < 0.001), in addition to having poorer sleep quality (*P* = 0.002). Those with lower cognitive scores were also more likely to be non-smokers (*P* < 0.001) or non-drinkers (*P* < 0.001), and they had significantly lower levels of social support (*P* < 0.001), lower body mass index values (*P* = 0.001), and lower annual household income (*P* = 0.001).

Table 2 shows that the cognitive ability scores of the frailty were also significantly lower than those of the non-frail (*P* < 0.001). Female frail participants had significantly lower C-MMSE scores than male frail participants (*P* < 0.001). As the number of debilitating components increased, C-MMSE scores decreased in both male and female older adults, and the differences were statistically significant (all *P* < 0.001).

**Table 2:** The physical frailty status and MMSE score in participants.

Variables	Total (*n* = 1654)			Men (*n* = 693)			Women (*n* = 961)		
	*n*, %	MMSE (M, Q_L_ ∼Q_U_)	*P*-Value^a^	*n*, %	MMSE (M, Q_L_ ∼Q_U_)	*P*-Value^a^	*n*, %	MMSE (M, Q_L_ ∼Q_U_)	*P*-Value^a^
Frailty	213(12.9)	19(15 ~ 22.5)	< 0.001	64(9.2)	22.5(17 ~ 25)	< 0.001	149(15.5)	18(14 ~ 21)	< 0.001
Robust	1441(87.1)	21(17 ~ 26)		629(90.8)	24(21 ~ 27)		812(84.5)	19(16 ~ 23)	
Components of the frailty									
Unintentional weight loss	177(10.7)	22(17 ~ 26)	0.387	73(10.5)	25(21 ~ 27)	0.346	104(10.8)	19(16 ~ 23)	0.584
Low grip strength	1401(84.7)	20(17 ~ 24)	< 0.001	506(73.0)	23(20 ~ 26)	< 0.001	895(93.1)	19(15 ~ 22)	< 0.001
Exhaustion	418(25.3)	20(16 ~ 24)	0.001	152(21.9)	24(21 ~ 26)	0.430	266(27.7)	18(15 ~ 22)	0.007
Low physical activity level	199(12.0)	20(16 ~ 24)	< 0.001	64(9.2)	24(20.5 ~ 26)	0.129	135(14.0)	18(14 ~ 21)	0.002
Slow gait speed	152(9.2)	17(13 ~ 20)	< 0.001	34(4.9)	20.5(16 ~ 24)	< 0.001	118(12.3)	16(12 ~ 19)	< 0.001
Components of the frailty			< 0.001			< 0.001			< 0.001
0	173(10.5)	26(22 ~ 28)		129(18.6)	26(24 ~ 28)		44(4.6)	23(21 ~ 27)	
1	869(52.5)	21(17 ~ 25)		379(54.7)	24(20 ~ 27)		490(51.0)	20(16 ~ 23)	
2	399(24.1)	20(16 ~ 24)		121(17.5)	24(20.5 ~ 27)		278(28.9)	18(15 ~ 22)	
≥ 3	213(12.9)	19(15 ~ 22.5)		64(9.2)	22.5(17 ~ 25)		149(15.5)	18(14 ~ 21)	

^a^Independent t-test for continuous variables and chi-square test for categorical variables.

**Fig. 1:**
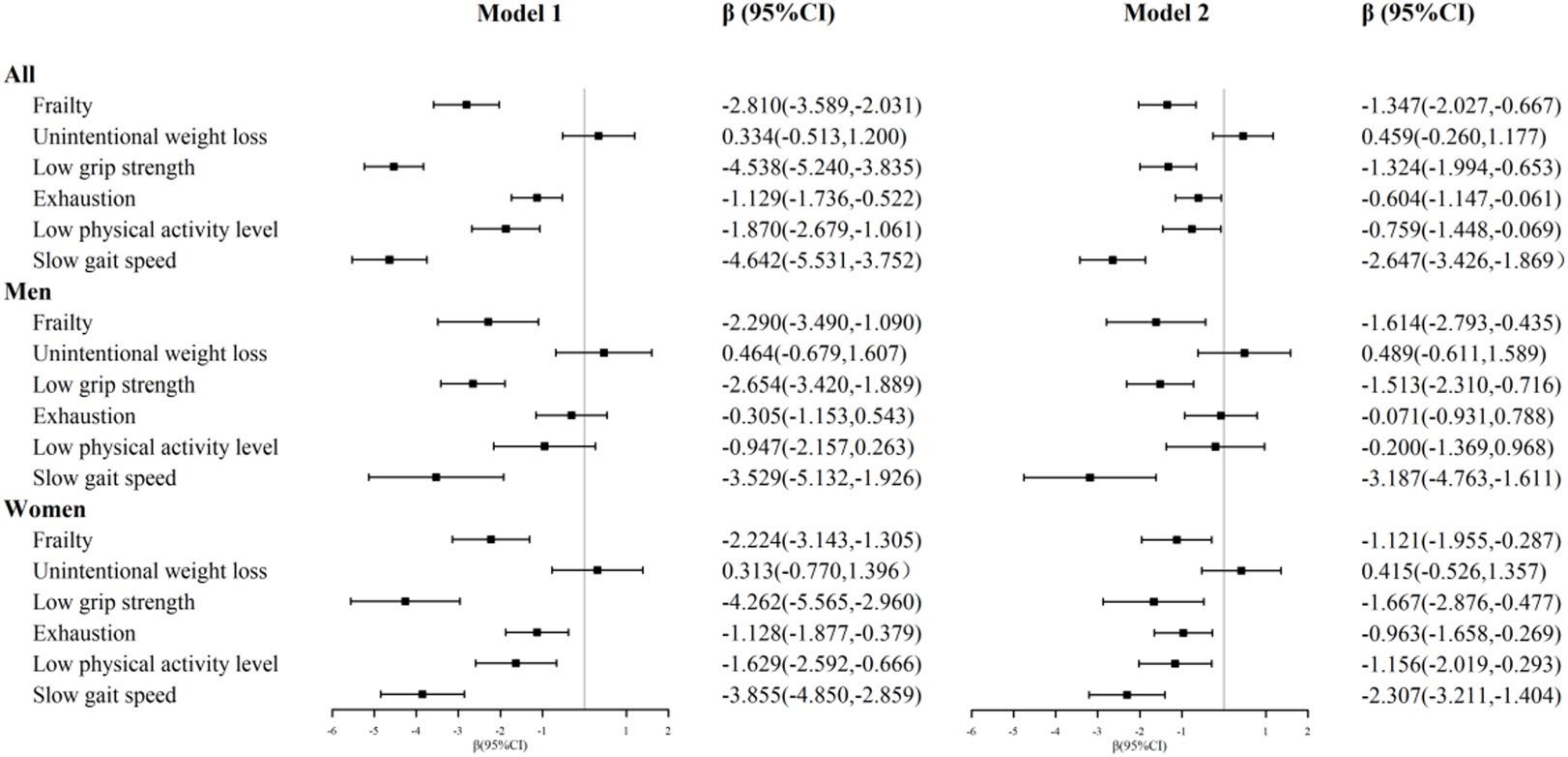
Relationships between C-MMSE scores and physical frailty in males and females. *Abbreviations*: CI: Confidence Interval. Model 1: Unadjusted. Model 2: Adjusted for age, gender, income, education, smoking, drinking, chronic disease, running, sleep quality, depression, social support level, and BMI other included unless the variable was used as a subgroup variable.

After adjustment for related variables, frailty was found to be significantly negatively associated with C-MMSE scores (*β*=−1.347, 95%CI: −2.027, − 0.667, *P* < 0.001). Consistently, C-MMSE scores were negatively associated with frailty in females (*β*=−1.121, 95%CI: −1.955, − 0.287, *P* = 0.008) and males (*β*=−1.614, 95%CI: −2.793, − 0.435, *P* = 0.007). All frailty phenotypic components other than unintentional weight loss were also negatively correlated with C-MMSE scores in females (All *P* < 0.05), whereas in males only low grip strength (*β*=−1.513, 95%CI: −2.310, − 0.716, *P* < 0.001) and slow gait speed (*β*=−3.187, 95%CI: −4.763, − 1.611, *P* < 0.001) were significantly related to C-MMSE scores (Fig. 1).

**Fig. 2:**
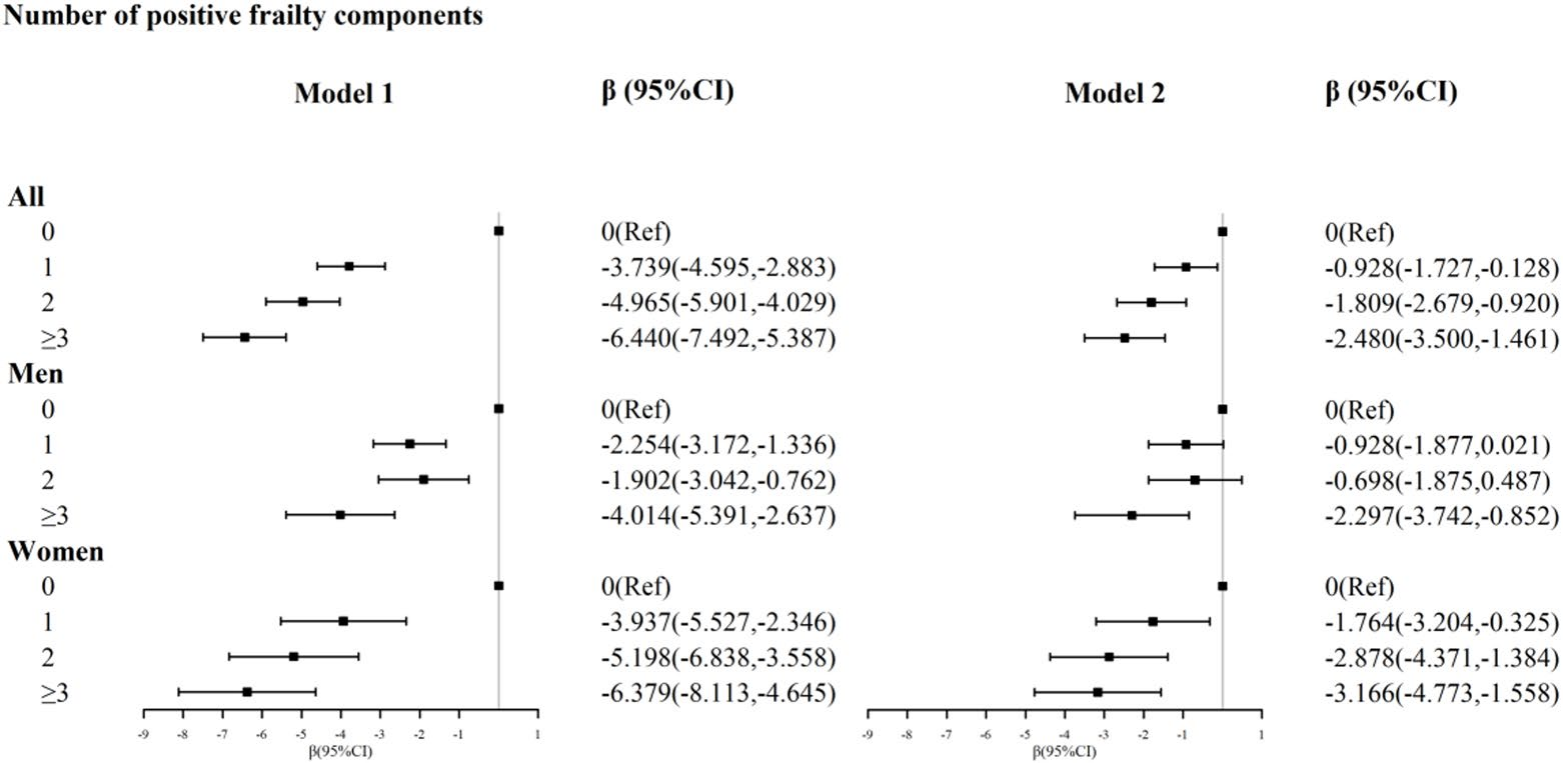
Relationships between C-MMSE scores and the number of positive frailty components for males and females. *Abbreviations*: CI: Confidence Interval. Model 1: No Adjustment. Model 2: Adjusted by age, gender, income, education, smoking, drinking, chronic disease, running, sleep quality, depression, social support level, and BMI were included unless the variable was used as a subgroup variable.

When assessing the cumulative number of the five components of frailty evident for each participant, the presence of 3 + total components of frailty was associated with lower C-MMSE scores among males (*β *= −2.297, *P* = 0.002), whereas the presence of just 1 + total components of frailty was associated with lower C-MMSE scores among females (*β *= −1.164, *P* = 0.016) after adjusting for age, education, income, smoking, drinking, chronic disease, exercise, sleep quality, depression, SSRS and BMI (Fig. 2).

## Discussion

In this cross-sectional analysis, significant associations were found between lower cognitive performance scores and low grip strength and slow gait speed in both men and women, whereas associations were found between cognitive function and fatigue or low physical activity only in women. The presence of 1 or more frailty components was associated with an elevated risk of cognitive decline in women, whereas the presence of 3 or more frailty components was associated with an elevated risk of cognitive decline in men. Thus, women with frailty face a higher risk of cognitive decline than men who experience frailty.

These analyses suggest that women tend to exhibit higher rates of frailty and lower C-MMSE scores compared to men, which may indicate that women’s health may be more susceptible to relevant factors. Several studies have also found that women are more likely than men to be physically frailty^28,29^. In one analysis of individuals with normal cognition, gender-related differences were observed with respect to brain aging such that females faced a higher risk of cognitive deterioration and Alzheimer’s disease^20,30^. Due to the interaction between frailty and cognitive decline^31,32^, resulting in older women being more susceptible to frailty and cognitive decline. This may be because women are more prone to frailty, which exacerbates cognitive decline, but more research evidence needs to be conducted to confirm this possibility.

Even after adjusting for potential confounders, the gender differences found in this study between C-MMSE scores and frailty components remained significant. Specifically, exhaustion and low physical activity were significantly associated with cognitive functioning only in women, which is consistent with previous research^30,33^. In general, women are more likely to exhibit exhaustion, low physical activity levels, unintentional weight loss, and lower grip strength relative to men^18,34^. Cognitive impairment has also been found to be associated with stress-related exhaustion, with a particularly strong relationship with delayed recall^35^. Some studies have shown that an increase in the number of frailty phenotypes at three and above is associated with an increased risk of cognitive impairment^36^. But in this study, women’s cognitive function was associated with any of the components of the frailty phenotype, whereas men’s cognitive functioning was associated with three or more of the frailty phenotypes. This suggests that women face a greater risk of cogitive decline than males due to frailty. To date, few studies have specifically explored gender-related associations between frailty and cognitive function. The gender differences in the association between frailty and cognitive functioning in the present study provide new ideas for future interventions to target the health of older adults. Early interventions targeting female frailty may also have a protective effect against cognitive decline, but this argument needs to be confirmed by future experimental studies.

The mechanistic basis for the gender differences between frailty and cognitive function remains unclear, but several factors may explain these results. Reduced testosterone production in older adults may contribute to this finding, with some studies pointing to lower levels of anabolic hormones, including free testosterone, as an independent predictor of frailty^37^. Several studies have shown that lower levels of testosterone and other androgens are associated with the risk of frailty and cognitive decline^38^. Lower testosterone levels in the male brain have also been linked to cognitive decline and a higher risk of developing Alzheimer’s disease^39^. Gender-related differences may also be influenced by the hypothalamic-pituitary-adr-.

enocortical axis (HPA), as dysfunction of the HPA axis can lead to aging-related disorders and neurocognitive dysfunction, including depression, cognitive deficits, and Alzheimer’s disease, as well as frailty due to altered levels of cortisol^40,41^.Older women are more susceptible than men to the development of cognitive decline and frailty due to altered cortisol levels, and cortisol plays an important role in the development of vulnerability to stressors in frail patients, with stressors increasing the risk of cognitive impairment, dementia, and depression in older adults. At the same time, the reasons for the observed gender differences may be related to genetic factors. For example, among older patients with mild cognitive impairment, ApoEε4 negatively affects memory and hippocampal performance more in females than in males^42^, the presence of certain ApoEε4 alleles also puts females at a significantly higher risk of developing Alzheimer’s disease than males^43^, and female ApoEε4 carriers have a higher rate of cognitive decline than men carrying the same gene^42^.

This study has several limitations. First, its cross-sectional design precludes causal inference regarding the relationship between frailty and cognition; prospective longitudinal studies are needed to validate these findings. Second, frailty was assessed using measures that retain a degree of subjectivity, which may introduce variability in classification and interpretation. Third, the sample was drawn from a single rural population, and external validation is required to determine the generalizability of the results to other settings and demographic groups. Finally, our cognitive assessment relied on the Chinese-adapted Mini-Mental State Examination (C-MMSE) without complementary neuroimaging, comprehensive neuropsychological testing, or biomarker data. As with the original MMSE, the C-MMSE has limited sensitivity for detecting subtle cognitive changes in older adults^44^. Future work should incorporate multi-domain neuropsychological batteries, alongside imaging and biomarker approaches, to provide a more granular characterization of cognitive function. Despite these limitations, the present study offers preliminary evidence that may inform future sex-specific, precision-oriented interventions.

In conclusion, there exist gender differences in the relationship between physical frailty and cognitive function in rural China, with women showing a stronger association. The results indicate that women’s cognitive function is more vulnerable to the effects of frailty; however, future prospective studies are needed to confirm this conclusion.

## Data Availability

No datasets were generated or analysed during the current study.
